# Complete Genome Sequence of *Escherichia*-Infecting Phage CEC_KAZ_2018, Isolated from Soil

**DOI:** 10.1128/MRA.00540-19

**Published:** 2019-09-05

**Authors:** Yergali S. Moldakhanov, Madina S. Alexyuk, Andrey P. Bogoyavlenskiy, Pavel G. Alexyuk, Aizhan S. Turmagambetova, Irina A. Zaitseva, Nadezhda S. Sokolova, Kuralay S. Akanova, Elmira I. Anarkulova, Elmira S. Omirtaeva, Vladimir E. Berezin

**Affiliations:** a Research and Production Center for Microbiology and Virology, Laboratory of Antiviral Protection, Almaty, Kazakhstan; Queens College

## Abstract

Avian pathogenic Escherichia coli (APEC) bacteria are one of the main problems of the poultry industry. An effective way to combat colibacillosis is to use a phage preparation that lyses the bacteria. Here, we report the isolation of an E. coli-infecting phage, CEC_KAZ_2018, isolated from soil.

## ANNOUNCEMENT

Escherichia coli is a Gram-negative, rod-shaped bacterium normally found in the intestines of poultry and most other animals. In poultry, as in humans, signs are nonspecific and vary with age, organs involved, and concurrent diseases. Economic impacts in broilers result from reduced growth, increased feed conversion rates, respiratory diseases, mortality, treatment cost, and condemnations, while in layers, losses are associated with decreased growth rates, mortality, and egg production. The problem is complicated by the emergence of antibiotic-resistant strains, necessitating a search for new approaches to the treatment of poultry. One of the solutions for the existing problem is to search for new phages capable of lysing pathogenic E. coli (1).

Here, we present the complete genome sequence of the CEC_KAZ_2018 bacteriophage, isolated from soil collected near one of the poultry farms near Almaty, Kazakhstan. Phage CEC_KAZ_2018 was detected to be using as its host the bacterium Escherichia coli, which was isolated from cecum broiler chickens. The bacterium was cultivated on MacConkey broth. Virulence factors associated with E. coli from broiler cecum and its classification as an avian pathogenic Escherichia coli (APEC) strain were not investigated in this study.

The genomic DNA was extracted from phage lysate of E. coli according to the guide for the PureLink viral DNA/RNA minikit (Thermo Fisher Scientific, USA). A Nanopore-compatible DNA library was prepared with SQK-LSK109 (Oxford Nanopore Technologies, Oxford, UK). The DNA library was sequenced using a SpotON flow cell (FLO-MIN106, R9), MinKNOW version 1.10.11, and the Albacore version 2.1.3 base caller in a 48-h sequencing protocol to generate 77,475 reads (2). The average, median, and *N*_50_ lengths of the raw Nanopore reads were 2,296 bp, 8,393 bp, and 2,935 bp, respectively. To analyze the data using Canu, all reads shorter than 1,000 nucleotides in length were removed, and a set of reads, which had total and *N*_50_ lengths of 4,573,439 bp and 2,935 bp, respectively, was received. A total of 13,043 reads were used for *de novo* assembly using Canu version 1.8 (3). The assembly generated a single contig of 48,562 bp. The quality of the genome assembly was determined by QUAST (4). The contig was analyzed with BLAST, the Genome Detective viral tool, and Geneious Prime 2019 (5–7). Detection of the physical ends of the viral genome was determined by comparing the coverage values of the length of the complete genome of the virus CEC_KAZ_2018 to those of closely related viruses in the NCBI database. The genome of the virus CEC_KAZ_2018 consists of 44,283 bp, with a GC content of 54.3%. Gene prediction was determined by GeneMark and PHAST (8–10). Sixty-five putative genes were predicted in the complete genome of CEC_KAZ_2018. The analysis of the phage open reading frames (ORFs) using a BLAST search showed that 27 ORFs correspond to proteins with a specific function. The remaining ORFs correspond to hypothetical viral proteins.

Therefore, the screening of soil should yield more phages that are genetically distinct and have therapeutic potential against bacterial infections caused by avian pathogenic E. coli.

### Data availability.

The complete genome sequence of *Escherichia* phage CEC_KAZ_2018 was deposited in GenBank under the accession number MK728541. Raw sequence reads are available under BioProject accession number PRJNA556777.
